# Langerhans Cell Histiocytosis Followed by Hodgkin Lymphoma: A Case Report

**Published:** 2015-05

**Authors:** Akbar Safaei, Mandana Bagheri, Jahanbanoo Shahryari, Sadat Noori, Elmira Esmailzade

**Affiliations:** 1 Department of Pathology, Molecular Pathology and Cytogenetic Ward, Shiraz University of Medical Sciences, Iran;; 2 Student Research Committee, Shiraz University of Medical Sciences, Shiraz, Iran

**Keywords:** Langerhans cell histiosis, Hodgkin disease, Immunohistochemistry

## Abstract

Langerhans cell histiocytosis (LCH) is a rare neoplasm defined as the proliferation of bone marrow langerhans cells, which is a kind of dendritic cells. The major pathological features of LCH are expression of CD1a and S100 as well as Birbeck granules. Its presentation can differ from a mild bone lesion to a multi-systemic evolved malignant neoplasm; however, the latter outcome is almost rare. Thus, LCH is mostly known as a benign neoplasm.

In this study, we present a case of LCH followed by Hodgkin lymphoma (HL). Accompaniment of this disease with malignant lymphoma is rare and considered as case report. Several cases in which malignant lymphoma occurred prior to LCH are reported; however, few cases can be found with LCH followed by malignant lymphomas.

## Introduction

Langerhans cell histiocytosis (LCH) is a rare neoplasm defined as the proliferation of bone marrow langerhans cells, which is a kind of dendritic cells.^1^^,^^2^ The major diagnostic features of LCH are expression of CD1a and S100 as well as ultrastructural Birbeck granules.^3^ Its presentation can differ from a mild bone lesion to a multi-systemic evolved malignant neoplasm; however, the latter outcome is almost rare. Thus, LCH is mostly known as a benign neoplasm.^2^

In this study, we present a case of LCH followed by Hodgkin lymphoma (HL). Accompaniment of this disease with malignant lymphoma is rare and considered as case reports. Several cases in which malignant lymphoma occurred prior to LCH are reported; however, few cases can be found with LCH followed by malignant lymphomas.^3^

## Case Report

In the present case report, we aim at describing an Iranian patient with langerhans cell histiocytosis followed by Hodgkin lymphoma. An informed consent was obtained from the patient for accessing his medical chart and the use of this data.

**Figure 1:**
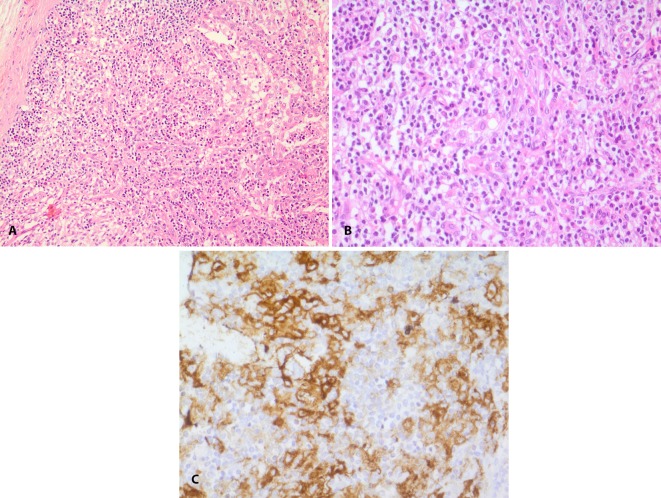
Langerhans cell histiocytosis. A &B: Infiltration of lymphocyte, plasma cells, eosinophils and many histiocytes with vesicular nucleus (H&E, x 200 & 400). C: Strong immunoreactivity of the histiocytes for CD1a (H&E, ×400)

A 23-year-old man was presented with generalized weakness, pain in lower extremities and an infiltrative mass in his right iliac bone. No lymphadenopathy or hepatosplenomegaly was detected. Laboratory data with a comprehensive metabolic panel, including complete blood count, activated partial thromboplastin time, liver function test, BUN, Cr, blood sugar were normal. C-reactive protein was 2^+^ and erythrocyte sedimentation rate was 21 mm h^-1^(after one hour). The chest radiograph was normal. Multiple axial, sagittal, and coronal MR images of the pelvic area were taken. The results demonstrated a hyperintense signal area in the right iliac bone with possible inflammation and/or infiltration. For further investigations, an excisional biopsy was provided. In histopathologic examination, in a background of lymphoplasma cells and eosinophils, many histiocytes with vesicular nucleus were detected. Immunohistochemistrical exams determined LCH in the mass by showing CD1a, the special marker of LCH (figure 1).

The patient responded to this surgical treatment, but one year later, an asymptomatic lesion was detected in a whole body bone scan, which revealed abnormal increase of signal in the right iliac bone, ischium, and superior side of acetabulum, which was assumed as tumoral infiltration.

Vinblastine and prednisone were given for the treatment of LCH, which were effective for about three years. During this period, the patient refused further followed up as he did not have any problem, but then, the disease relapsed.

**Figure 2:**
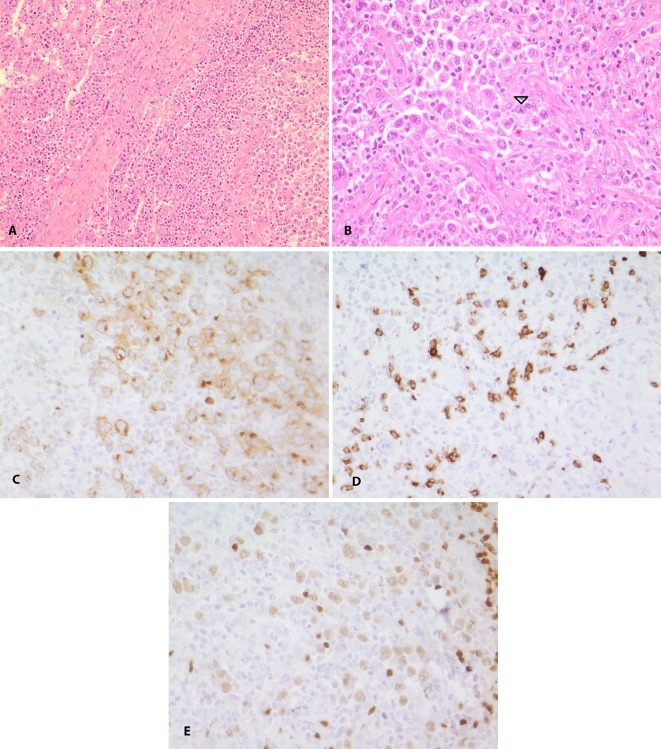
A) Low power view of Hodgkin lymphoma at the right side and langerhans cell histiocytosis at the left side (H&E, ×200). B) High power view of Hodgkin lymphoma with many Reed-Sternberg cells (arrow). These cells have mono and binucleated vesicular nucleus with conspicuous nucleoli (H&E, ×400). C-E: Immunoreactivity of Reed-Sternberg cells with CD30, CD15, and weak staining with PAX5 (H&E, ×400)

At the age of 28, he developed inguinal lymphadenopathy (LAP) as well as a poorly defined lesion in his pelvic inlet. Color-Doppler ultrasonography of both lower extremities, arteries, and deep veins showed multiple enlarged lymph nodes in both inguinal and femoral triangles; diffuse infiltrative hypoechoic lesions in central retro peritoneum, around the abdominal aorta and inferior vena cava were also noted suggestive of diffuse retroperitoneal fibrosis. Biopsy of one side was done. Histopathology and immunohistochemistrical studies of inguinal LAP revealed a composite picture. It showed nodular pattern of mixed cellularity type of Hodgkin lymphoma characterized by background of eosinophils, plasma cells and lymphocytes, some Reed-Sternberg cells (classic or mononuclear forms) with prominent large eosinophilic nucleoli (positive for CD15, CD30 and weakly PAX5, negative for S100, CD1a, CD20 and CD45), accompanied by LCH with diffuse neoplastic proliferation of Langerhans cells in other part (figure 2). Previous slides of patient’s lesion were reviewed, confirming the diagnosis of LCH. Thus, morphologic and IHC findings confirmed the diagnosis of Hodgkin Lymphoma following LCH.

For treatment, he was on ABVD (Adriamycin, Bleomycin, Vinblastine, and Dacarbazine) as a chemotherapy regimen. After a few courses of chemotherapy, PET/CT scan showed good response to the treatment. 

## Discussion

Langerhans cells are derived from bone marrow dendritic cells and show pale clefted nuclei, abundant pale eosinophilic cytoplasm, and Birbeck granules. It has some properties with macrophages and monocytes, but differ from other histiocytes as they are positive for CD1a.^3^

Generally, LCH can be found as a primary disease ranging from solitary bone lesions to polysystemic disease or it may present with a diverse clinical manifestation, such as diabetes insipidus.^4^ Moreover, in most cases, it is considered as a benign disorder that rarely ends in malignant phenomenon.^2^ Hodgkin lymphoma is a B-cell lymphocyte neoplasm which is specifically associated by Hodgkin-Reed-Sternberg (HRS) cells, which by the way are absent in LCH.^5^ Eosinophil is also merely found in LCH. Therefore, these two characteristics can be considered as differential pathologic features.^6^

The occurrence of lymphoma and LCH in the same individual, either sequentially or simultaneously is not common. Moreover, the association of LCH and Hodgkin Lymphoma is rare.^7^

The relationship between LCH and HL is still unclear and is just discussed as a case report. Greaves et al. reported the simultaneous presence of LCH and HL in a patient undergoing multi-agent chemotherapy.^8^ Moreover, Hyun et al. were also reported a concurrent occurrence of LCH and HL of spine in a patient and declared it as a rare condition.^9^ Also, in another study conducted by Régis et al., two cases were reported to have both LCH and malignant lymphomas.^10^ In other studies, more patients were described for concomitance of these two disorders. In a report by Egeler et al., 39 patients were found to have LCH and malignant neoplasm, among them, there were 25 who had LCH and HL concurrently. In 10 patients, lymphoma preceded LCH , and only in 4 patients the diagnosis of LCH preceded lymphoma by 6-24 months later, in which the type of lymphoma in 3 of them was non-Hodgkin lymphoma and only one of the cases had HL.^2^ Furthermore, a study by Dehkordi et al. reported on a 10-year-old boy who was diagnosed by LCH three years after the treatment and diagnosis of Hodgkin lymphoma.^11^ As an explanation, it was declared that malignant lymphomas may be able to induce the proliferation of abnormal Langerhans cells which leads to LCH.^3^ A kind of response to Hodgkin lymphoma microenvironment is abnormal neoplastic proliferation of stromal cells. Smaller size of these cells makes differential diagnosis more difficult.^7^

Hodgkin lymphoma should be considered in the differential diagnosis of recurrent LCH. Distinguishing the two diseases is very crucial because of their far different management. Immunohistochemistry can be helpful in such circumstances.

As it is observed, in most cases, LCH emerged concurrently or after the lymphoma; however, few cases can be found in which LCH developed before the malignant lymphomas. In 2012, one case in Korea was reported with LCH followed by HL.^3^

In this study, we introduced a patient in whom LCH was diagnosed and 5 years later HL was seen as well. Although some physicians may assume that these two disorders may have occurred concurrently, one was not diagnosed. We reevaluated previous slides and our presented case responded to LCH therapy alone for 3 years. Therefore, the possibility of concurrence with HL can be put aside.

Long interval between the development of HL secondary to LCH suggests an association between these two diseases without relation to chemotherapy treatments.

Therefore, it is important to consider the probability of occurrence of these two conditions in the same patient and using appropriate technique to differentiate them to avoid misdiagnosis. An accurate diagnosis in such situations helps the physician to choose appropriate therapy. 
